# New generation of two-dimensional spintronic systems realized by coupling of Rashba and Dirac fermions

**DOI:** 10.1038/srep12819

**Published:** 2015-08-04

**Authors:** Sergey V. Eremeev, Stepan S. Tsirkin, Ilya A. Nechaev, Pedro M. Echenique, Evgueni V. Chulkov

**Affiliations:** 1 Institute of Strength Physics and Materials Science, 634021, Tomsk, Russia; 2 Tomsk State University, 634050, Tomsk, Russia; 3 Donostia International Physics Center (DIPC), 20018 San Sebastián/Donostia, Basque Country, Spain; 4 Saint Petersburg State University, Saint Petersburg, 198504, Russia; 5 Departamento de Física de Materiales UPV/EHU, Facultad de Ciencias Químicas, UPV/EHU, Apdo. 1072, 20080 Sebastián/Donostia, Basque Country, Spain; 6 Centro de Física de Materiales CFM - MPC, Centro Mixto CSIC-UPV/EHU, 20080 San Sebastián/Donostia, Basque Country, Spain

## Abstract

Intriguing phenomena and novel physics predicted for two-dimensional (2D) systems formed by electrons in Dirac or Rashba states motivate an active search for new materials or combinations of the already revealed ones. Being very promising ingredients in themselves, interplaying Dirac and Rashba systems can provide a base for next generation of spintronics devices, to a considerable extent, by mixing their striking properties or by improving technically significant characteristics of each other. Here, we demonstrate that in BiTeI@PbSb_2_Te_4_ composed of a BiTeI trilayer on top of the topological insulator (TI) PbSb_2_Te_4_ weakly- and strongly-coupled Dirac-Rashba hybrid systems are realized. The coupling strength depends on both interface hexagonal stacking and trilayer-stacking order. The weakly-coupled system can serve as a prototype to examine, e.g., plasmonic excitations, frictional drag, spin-polarized transport, and charge-spin separation effect in multilayer helical metals. In the strongly-coupled regime, within ~100 meV energy interval of the bulk TI projected bandgap a helical state substituting for the TI surface state appears. This new state is characterized by a larger momentum, similar velocity, and strong localization within BiTeI. We anticipate that our findings pave the way for designing a new type of spintronics devices based on Rashba-Dirac coupled systems.

Over the past few years, great attention is paid to 2D electron systems that hold helical (spin-momentum locked) electron states induced by spin-orbit interaction (SOI), such as giant-Rashba-split states or topological Dirac states^1^,^2^,^3^,^4^,^5^. Suitable Dirac systems are already routinely observed on surfaces of three-dimensional topological insulators. The main concern here is that the corresponding 2D backscattering-protected helical states are desirable to be energetically and spatially well separated from TI bulk states. It is seen as a way to refine on their remarkable characteristics, be it, e.g., the helical spin-polarized transport^6^,^7^,^8^,^9^,^10^,^11^,^12^ or the Dirac plasmon, which carry spin^13^,^14^ and, consequently, may lead to charge-spin separation effects expected for thin (about 100 nm) films of a TI^15^.

Until recently, Rashba systems, which were mainly associated with 2D electron gases of semiconductor heterostructures and as such were extensively studied, e.g., in the context of spin-polarized transport, are inferior to Dirac systems, first and foremost, because of the very small magnitude of the SOI term. Now, the semiconductors BiTe*X* (with *X* = Cl, Br, I) came into focus, because of the sizeable Rashba-type spin splitting of their bulk and surface states^4^,^5^,^16^,^17^,^18^,^19^,^20^, arising from a strong SOI and the material polarity. In this case, the SOI term is not a weak perturbation with respect to the band kinetic energy anymore.

A promising combination of the 2D Rashba system formed by surface-state electrons of the BiTe*X* surface and the graphene Dirac system has been theoretically proposed in Ref. [21], though in this case original graphene Dirac state has no spin-momentum locking. To involve 2D helical Dirac states, one should treat a semi-infinite TI or a TI film as thick as about 10 its structure elements. Thus, we have a situation, when both desired Rashba and Dirac electron systems are provided by surfaces.

However, in contrast to 2D spin-helical Dirac fermions, the 2D Rashba-fermion system with giant spin-orbit splitting can be represented merely by a structure element of BiTe*X*. Owing to three-layered (TL) structure of these compounds with the *X*-Bi-Te stacking within the TL, an ultrathin film of one-TL thickness can be exfoliated from the bulk crystal or grown epitaxially on suitable substrate. Among BiTe*X*, it is BiTeI, which provides us with the biggest Rashba interaction strength *α*_*R*_ ≈ 1.6 eVÅ, as calculated within density functional theory (DFT) (see details in Supplementary Section S1) for the conduction band of a single TL possessing the band gap of 750 meV [see Fig. 1(a)]. Such a strength is half as much as compared to the Te-terminated surface state of BiTeI, but it is still an order of magnitude greater than that in the conventional semiconducting heterostructures.

A desired interplay of Dirac and Rashba fermions can thus be realized by depositing a BiTeI TL on top of a TI. The most geometrically suitable TI in this case is PbSb_2_Te_4_^22^, whose in-plane hexagonal parameter matches perfectly with the parameter of BiTeI. This TI has a 220 meV calculated indirect band gap hosting the spin-helical Dirac surface state [see Fig. 1(b)]. As seen in Fig. 1(c) showing the electronic spectrum of a system composed of *non-interacting* TL and TI, energetically we have the fortunate alignment of the Dirac and Rashba bands within the projected band gap of the bulk PbSb_2_Te_4_; the degeneracy point of the Rashba state lies just a few meV above the Dirac point. Regarding the helicity of the Rashba and Dirac states, in the upper Dirac cone and in the outer Rashba branch the spin locked to the momentum can be in-plane polarized in the same or opposite directions, depending on how the TL is oriented relative to the TI. In that sense, we have two TI-TL interfaces: Te-I (hereafter marked as TII) with the same directions [as shown in Fig. 1(c)] and Te-Te (below we refer to it as to TTI) with the opposite directions. Thus, one can expect that the *interacting* picture should vary with the type of interface.

In order to treat the TI-TL interaction properly, we have optimized interface and interlayer distances within the TL for both interfaces with different hexagonal stacking (A-A or A–C, see Fig. 2(a–d)). We have found only a very small difference between their total energies (see Supplementary Section S1), so that both types of the interfaces can be realized at suitable growth conditions. In the BiTeI@PbSb_2_Te_4_ heterostructure with Te-Te and Te-I interfaces, the relative position of the Dirac and Rashba states will apparently depend on the interface coupling (see also Supplementary Section S1).

The layer- and spin-resolved electron energy spectra for both interfaces of the BiTeI@PbSb_2_Te_4_ heterostructure are shown in Fig. 2. The TTI-AC [Fig. 2(e)] does not give evidence of any notable effect of the coexistence of the Rashba and Dirac subsystems. The point is that the local layer stacking at the TTI-AC between the Te-atomic layers, being the same as that between van-der-Waals bonded septuple layers (SL’s) in the bulk PbSb_2_Te_4_, and the subsequent atomic layers of the TL prolong effectively this very TI, pulling partly the surface state into BiTeI (Fig. 2(i)). The resulting surface energy spectrum within the projected bulk band gap resembles that of the pristine PbSb_2_Te_4_ with a slightly increased Dirac velocity. Figure 2(f) demonstrates that, as compared with the non-interacting case presented in Fig. 1(c), at the TTI-AA the Dirac state remains practically intact, but the Rashba state is slightly shifted up so that its degeneracy point lies 83 meV above the Dirac point. Both 2D states retain its spin polarization and spatial localization inherent to the free-standing TL and the pristine TI surface. Figure 2(j) demonstrates quite well spatial separation of the Rashba and Dirac states. We also note that the crossings of the Rashba and Dirac bands with different helicity are not avoided.

Substantially different picture is observed in the case of the TII [Fig. 2(g,h)]. First, by changing the stacking order in the TL (from Te-Bi-I to I-Bi-Te), we reverse the direction of the in-plane spin polarization for the Rashba branches. Second, owing to the interface potential bending (see Supplementary Section S1), the states of TI move up and the states of BiTeI move down resulting in that now the former degeneracy point of the Rashba state lies below the former Dirac point. Upon moving from the TII-AA (Fig. 2(g)) to the TII-AC (Fig. 2(h)) the coupling of Dirac and Rashba fermions becomes stronger and, as shown below, the energy distance between these points increases mainly due to the Dirac point shifting up. Third, owing to avoided crossing of the bands with the same helicity, a gap opens up (highlighted by a yellow stripe in Fig. 2). As a result, below the bulk conduction band emerges a Rashba-like state derived from the inner branch of the former TL-localized Rashba state and the upper Dirac cone. This electron-like Rashba state has the opposite spin helicity in the inner and outer branches. The inner branch is localized in the topmost SL of PbSb_2_Te_4_ and the outer branch is localized in the TL (Fig. 2(k,l), green and cyan lines, respectively). The lower part of the former Dirac state transforms into a hole-like outer Rashba branch resided just above the top of the TI valence band.

The most prominent feature of the TII spectrum is the gap near the Fermi level, which is of ~100 meV in the case of the A–C interface stacking [Fig. 2(h), yellow stripe]. In this gap, as well as in the pristine TI surface, we have a backscattering-protected helical state only. However, there are various advantages over the TI. Actually, this state originated from the outer branch of the former Rashba state is strongly localized within the BiTeI TL (Fig. 2(l), pink line), conforming to the 2D nature to a greater extent than the TI surface state does. Due to such a localization, the dielectric screening of the Coulomb interaction between 2D helical fermions caused by surrounding medium becomes notably weaker (the dielectric constant *ε* ≈ (*ε*_TI_ + *ε*_Vacuum_)/2, where *ε*_TI_ = 53 is the dielectric constant of PbSb_2_Te_4_ as found in Supplementary Section S3). Additionally, within the energy interval corresponding to the gap the helical Rashba-derived state is characterized by the velocity similar to the Dirac state on the pristine PbSb_2_Te_4_ surface, while the momentum and the density of states are several times higher. Further, we will focus on the TTI-AA and TII-AC interfaces only.

In order to investigate single-particle and collective excitations in the interfaces under study, we construct a model Hamiltonian that correctly reproduces the *ab initio* results on the dispersion and spin texture of the respective 2D states. The model Hamiltonian reads


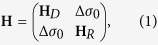


where the parameter Δ determines the coupling between the Dirac (*D*) and Rashba (*R*) 2D subsystems, allowing for hybridization between states of the same helicity only. In Eq. (1), the Fu Hamiltonian **H**_*D*_ (without the warping term)^23^ and the Rashba Hamiltonian **H**_*R*_^24^ have the form diagonalized by the rotation in spin space generated by *U*_***k***_ = exp[*iπ*(***σ*** ⋅ **k**)/(4*k*)] (see e.g. Ref. [25]), i.e., 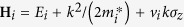. Here, *i* runs over the subsystems *D* and *R*, the energies *E*_*i*_ set the positions of the Dirac and Rashba degeneracy points, *σ*_0_ and ***σ*** = (*σ*_*x*_, *σ*_*y*_, *σ*_*z*_) are the Pauli matrices, *v*_*D*_ = *v*_*D*0_(1 + *γk*^2^) is the Dirac velocity with a second-order correction, and *v*_*R*_ ≡  *α*_*R*_ is the strength of the Rashba spin-orbit interaction.

The Hamiltonian (1), describing the resulting Dirac-Rashba hybrid electron system, is diagonalized, 
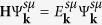
, with energy bands given by


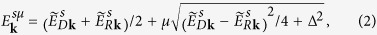


where *s*, *μ* = ±1 and 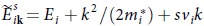, and with respective wavefunctions 
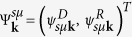
 composed of two-component spinors 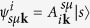, where the normalization constant 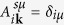 at Δ = 0. In real space, the wavefunctions have the desired form of 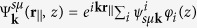 with 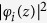 characterizing the localization of the corresponding subsystem in the direction *z* perpendicular to the surface plane. Values for all the model parameters mentioned above are found by fitting the *ab-initio* dispersions in the coupled regime (see Supplementary Section S2). Within such a model description, we end up with the layer- and spin-resolved band structure shown in Figs 3,4. As follows from the figures, the main features of the *ab-initio* spectra presented in Fig. 2(h,f) are nicely reproduced within our model with the proper relative alignment of the Dirac and Rashba degeneracy points. In the case of the TII-AC, because of an avoided crossing of the bands with the same helicity, we have a “gap” of ~100 meV (see the yellow stripe in Fig. 3), where a backscattering-protected helical states appears to be localized predominantly in the Rashba subsystem. In the case of TTI-AA, where only the bands with different helicity cross, a superposition of slightly modified original bands with some mutual shift of the degeneracy points is simulated.

Now, we can take advantages offered by the model in description of single-particle and collective excitations at the interfaces under study. With the energy bands and the respective wavefunctions, we can construct the non-interacting response function of the Dirac-Rashba hybrid electron system, and further, within random phase approximation, the interacting response function can be derived (see details in Supplementary Section S2). In Fig. 3, we present our results by plotting the electron energy loss function *g*(**q**, *ω*) for the TII-AC. As far as the Fermi energies can be routinely tuned within the bandgap, we considered three distinct Fermi-level positions (see Fig. 3). First, we note that for all the considered Fermi-level positions in the pure Dirac system the plasmon spectrum goes very close to the border of the intraband Landau damping region, owing to a large dielectric constant of PbSb_2_Te_4_. Moreover, the Dirac plasmon free of Landau damping (just with a quite small linewidth due to a finite broadening related to a typical value of the relaxation time *τ*_*r*_ ~ 500 fs^26^) appears in far-infrared region, where only optical mode of a TI-thin slab was detected by infrared spectroscopy^14^. Further, the plasmon enters into the mentioned damping region and acquires a finite and rapidly diminishing lifetime. With decreasing 2D charge density (*E*_*F*_ = −0.01 eV), the plasmon tends to disappear.

The pure Rashba system, where, nevertheless, 2D electron-electron interaction is modified by the presence of the TI substrate, is characterized by the plasmon spectrum that goes quite far from the border of the intraband damping region (Fig. 3). For *E*_*F*_ = 0.08 eV and 0.20 eV, the undamped plasmon already reaches long-infrared region and just at excitation energies *ω* ~ 0.11 eV and higher hits the border of the SOI-induced interband damping region, whereupon Landau damping occurs. For *E*_*F*_ = −0.01 eV, the wedge, where the plasmon is free of Landau damping, is very small, and, as a consequence, a finite plasmon lifetime is observed already at *ω* ~ 0.01 eV.

As seen in Fig. 3, the Dirac-Rashba hybrid system shows a variety of possible plasmon spectra and the electron-hole-continuum edges generated by shifting the Fermi level. At the Fermi energy above the gap (*E*_*F*_ = 0.20 eV), as a consequence of the presence of two interacting electron subsystems, two modes appear: the optical and acoustic mode. The optical plasmon touches the interband damping region at *ω* ~ 0.07 eV, while the acoustic plasmon is damped, since it lies entirely in the intraband damping region. The wedge with the base attached to the *ω* interval from ~0.12 eV to ~0.18 eV is caused by the presence of the gap. On the whole, the dispersion of the plasmons is merely slightly changed as compared with the Δ = 0 case, when the subsystems are presented by the bands shown by dashed lines in Fig. 3 and coupled only electrostatically.

The most attractive case with the position of the Fermi level in the middle of the gap (*E*_*F*_ = 0.08 eV) is characterized by a plasmon branch that lies rather far from the intraband damping region and enters into the region of interband transitions at *ω* ~ 0.10 eV. In this case, the plasmon is free of Landau damping within a quite large part of the *ω* − *q* plane under study. It turns out that by depositing the TL of BiTeI the Dirac plasmon of the pristine PbSb_2_Te_4_ is “pulled out” (see the corresponding loss function for the pure Dirac system) into the long-infrared region.

At the Fermi level below the gap (*E*_*F*_ = −0.01 eV), again the characteristic of two interacting electron subsystems giving two oscillation modes reads clearly. The interesting feature here is the presence of a quite wide *ω* interval (from ~0.06 eV to ~0.14 eV), where, owing to the presence of the gap, for relatively big *q* the optical plasmon has rather small linewidth. Thus, by tuning the Fermi level, one can single out a preferred region of the *ω* − *q* plane, where the collective excitations are well defined.

Figure 4 shows the electron energy loss function modeled for the TTI-AA. Here, in the case of the pure Rashba system the Rashba branches are shifted up as they appear in the weakly-coupled regime in order to simplify a comparison with the latter. As a result, it is easily seen that both the plasmon spectrum and the electron-hole continuum, which we have for the Dirac-Rashba hybrid system here, resemble those of merely electrostatically coupled electron subsystems with a damped acoustic plasmon. This means that the TTI-AA can serve as a prototype providing us with a coexistence of Dirac and Rashba fermions, which are well separated without need to use a spacer. Consequently, a simplified theoretical description of the real system can be applied in order to, e.g., probe the Dirac plasmon by examining the optical mode, to study the frictional drag, to analyze possible charge-spin separation effects, etc. (see, e.g., Refs. [15,^27^,^28^]).

Besides showing the loss function *g*, in Figs 3,4 we plot the real part *σ*_1_ of the optical conductivity (see Supplementary Section S2), which is practically useful, since it gives an idea about propagation of the plasmon modes. In all cases, at the *ω* → 0 limit the real part has a Drude peak caused by the mentioned finite relaxation time introduced. The figures clearly demonstrate that in the resulting optical conductivity *σ*_1_ the respective Rashba contribution dominates. Nevertheless, there are several extra features caused by inter-subsystem coupling, which also make the hybrid systems practically attractive. For the TII-AC considered in Fig. 3, in the case of *E*_*F*_ = 0.20 eV additional interband transitions notably increase *σ*_1_ for the excitation energy *ω*  0.07 eV, while for the interval from 0.12 eV to 0.18 eV *σ*_1_ is reduced to zero on account of the presence of the gap in the band structure of the hybrid system. At *E*_*F*_ = 0.08 eV, again due to the gap a quite pronounced peak effectively decreasing the plasmon propagation length appears around *ω* ~ 0.10 eV. With *E*_*F*_ = −0.01 eV, on the contrary we see the formation of a gap of ∼0.08 meV in *σ*_1_. Such a gap cuts out the long-infrared region, where the plasmon with rather big momenta has a quite long lifetime. For the TTI-AA, *σ*_1_ can be well approximated by a sum of the real parts *σ*_1_ of the pure Dirac and Rashba subsystems.

Finally, to go further in our study of excitations in the proposed interfaces, we derived a proper expression for the inelastic decay rate Γ within the *G*_0_*W*_0_ approximation. For the case of *E*_*F*_ = 0.08 eV, we calculated the decay rate for quasiparticles in the helical states, which reside within the energy interval (*E*_*F*_ ± 0.04 eV) corresponding to the yellow stripe shown in Fig. 3 (see Supplementary Section S2). In the pure Dirac system, these states belong to the upper Dirac cone, while in the Dirac-Rashba hybrid system we treat the Rashba-derived states. The calculations showed that within the mentioned energy interval in the hybrid system the inelastic decay rate is slightly smaller for excitation energies 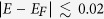 eV and tends to be somewhat bigger beyond this range, as compared with respective Γ in the pure Dirac system. On the whole, the resulting Γ in the hybrid system is surprising to be well approximated by the functional dependence of the decay rate on *ω* as was already found for Rashba fermions^29^. Actually (see Supplementary Section S2), the respective decay rate presented as Γ/*E*_*F*_ ∝ *af*(*ξ*) with *ξ* = *ω*/*E*_*F*_ is nicely reproduced by *f*_*R*_(*ξ*) = −*ξ*^2^[ln (*ξ*/16) − 1/2 − *γ*ln (*γ*/8)]/(4*π*) all over the examined energy interval with the constant of proportionality *a* = 0.61 for electrons and *a* = 0.73 for holes (here, *γ* = *E*_*α*_/*E*_*F*_ with the Rashba energy 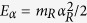). In the case of the pure Dirac system, the dependence *f*_*D*_(*ξ*) = −*ξ*^2^[ln (*ξ*/8) + 1/2]/(4*π*) can be used^30^. However, in order to cover all the excitation-energy interval the term −*ξ*^2^/8 should be added to *af*_*D*_(*ξ*) with *a* = 1.04 for electrons and *a* = 1.17 for holes. On the whole, the lifetime of quasiparticles in the Dirac-Rashba hybrid system is as long as ~0.2 ps at the border of the energy interval under study.

## Additional Information

**How to cite this article**: Eremeev, S. V. *et al.* New generation of two-dimensional spintronic systems realized by coupling of Rashba and Dirac fermions. *Sci. Rep.* **5**, 12819; doi: 10.1038/srep12819 (2015).

## Supplementary Material

Supplementary Information

## Figures and Tables

**Figure 1:**
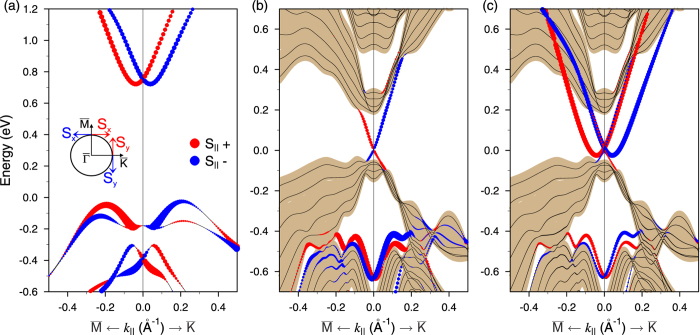
Spin-resolved electronic structure (red and blue colors denote positive and negative values of in-plane spin, respectively, for mutually perpendicular 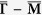 and 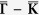 directions) of (**a**) free-standing BiTeI trilayer (TL); (**b**) PbSb_2_Te_4_ surface; (**c**) the system of BiTeI TL at distance of ≈7 Å above the PbSb_2_Te_4_ surface modeled by a six septuple-layer (SL) slab. Projected bulk band structure of PbSb_2_Te_4_ marked by brown areas.

**Figure 2:**
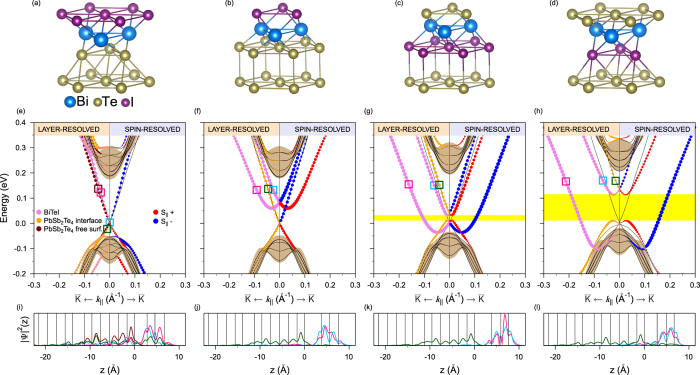
Interface atomic structure and layer- and spin-resolved electronic structure of BiTeI@PbSb_2_Te_4_: Te-Te interface with the A–C (**a**,**e**) and A-A (**b**,**f**) hexagonal stacking; Te-I interface with the A-A (**c**,**g**) and A–C (**d**,**h**) hexagonal stacking. In panels (**a**–**d**) the lowest Te layer is surface atomic layer of PbSb_2_Te_4_ and next three layers is BiTeI TL. In panels (**e**–**h**) blue and red filled circles denote the clockwise and anti-clockwise spin-momentum locking, respectively, and the violet, orange and brown filled circles denote the localization on the BiTeI TL, the PbSb_2_Te_4_ interface SL and the PbSb_2_Te_4_ free-surface SL, respectively. Spatial distribution of the charge density integrated over *x*, *y* planes, 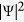, for localized states within the gap: Te-Te interface with the A–C (**i**) and A-A (**j**) hexagonal stacking; Te-I interface with the A-A (**k**) and A–C (**l**) hexagonal stacking; color of lines corresponds to the squares marked in panels (**e**–**h**).

**Figure 3:**
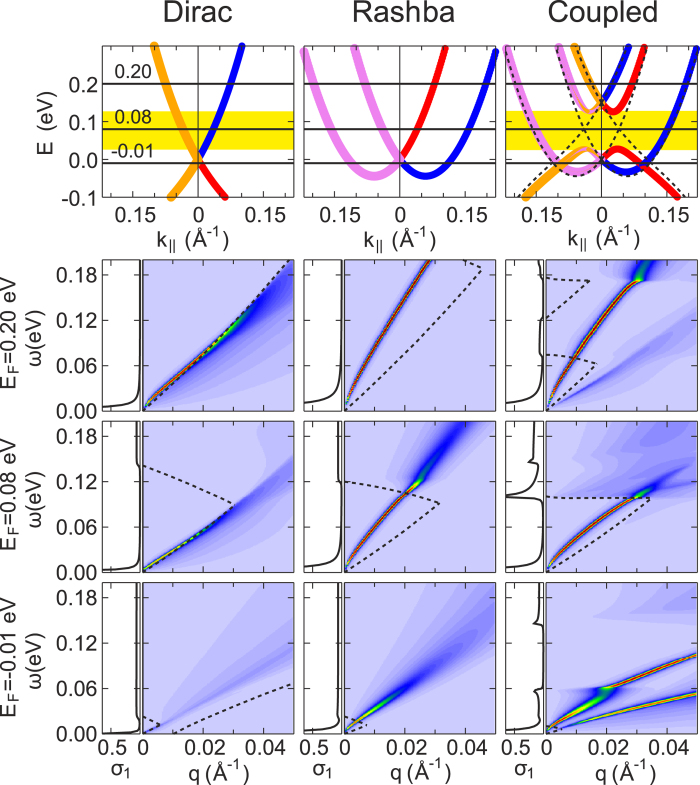
Upper row: Layer- and spin-resolved band structure of the pure Dirac (left) and Rashba (center) subsystems taken separately [as they appear in Fig. 1(c)], as well as the Dirac-Rashba hybrid system in the strongly-coupled regime (right) imitating the TII-AC shown in Fig. 2(h). Here, the blue (red) color denotes the clockwise (anti-clockwise) spin-momentum locking and the violet (orange) color denotes the localization on the Rashba (Dirac) subsystem. Dashed lines show the band structure of the Dirac-Rashba system in the decoupled regime (Δ = 0). **Lower rows:** The respective color maps of the electron energy loss function *g* and graphs of the real part *σ*_1_ (in 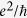) of the optical conductivity as obtained for different Fermi energies indicated in the upper panels. Dashed lines show the borders of the particle-hole continuum.

**Figure 4:**
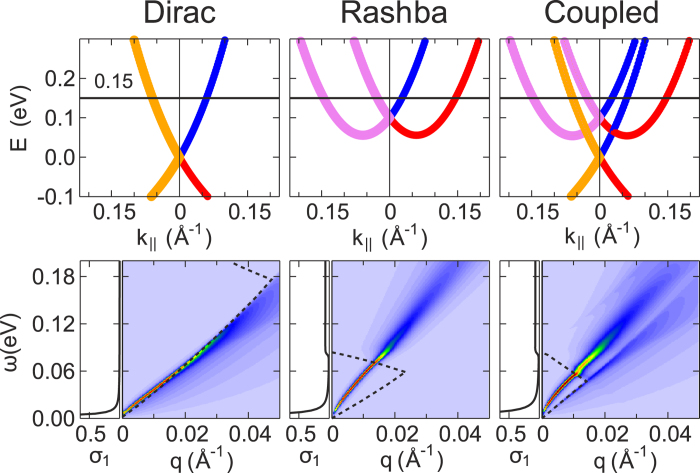
Same as in Fig. 3 but for the TTI-AA and for one position of the Fermi level *E*_*F*_ = 0.15  eV. Here, as compared with Fig. 1(c) the pure Rashba band is upshifted to coincide with that in the weakly-coupled regime.

## References

[b1] Hasan M. Z. & Kane C. L. Colloquium: Topological insulators. Rev. Mod. Phys. 82, 3045 (2010).

[b2] Qi X.-L. & Zhang S.-C. Topological insulators and superconductors. Rev. Mod. Phys. 83, 1057 (2011).

[b3] Sau J. D., Lutchyn R. M., Tewari S. & Das Sarma S. Generic New Platform for Topological Quantum Computation Using Semiconductor Heterostructures. Phys. Rev. Lett. 104, 040502 (2010). 20366693 10.1103/PhysRevLett.104.040502

[b4] Ishizaka K. *et al.* Giant Rashba-type spin splitting in bulk BiTeI. Nature Materials 10, 521 (2011). 21685900 10.1038/nmat3051

[b5] Eremeev S. V., Nechaev A. I., Koroteev Y. M., Echenique P. M. & Chulkov E. V. Ideal Two-Dimensional Electron Systems with a Giant Rashba-Type Spin Splitting in Real Materials: Surfaces of Bismuth Tellurohalides. Phys. Rev. Lett. 108, 246802 (2012). 23004307 10.1103/PhysRevLett.108.246802

[b6] Burkov A. A. & Hawthorn D. G. Spin and Charge Transport on the Surface of a Topological Insulator. Phys. Rev. Lett. 105, 066802 (2010). 20867997 10.1103/PhysRevLett.105.066802

[b7] Yazyev O. V., Moore J. E. & Louie S. G. Spin Polarization and Transport of Surface States in the Topological Insulators Bi_2_Se_3_ and Bi_2_Te_3_ from First Principles. Phys. Rev. Lett. 105, 266806 (2010). 21231702 10.1103/PhysRevLett.105.266806

[b8] Li C. H. *et al.* Electrical detection of charge-current-induced spin polarization due to spin-momentum locking in Bi_2_Se_3_. Nature Nanotechnol. 9, 218 (2014). 24561354 10.1038/nnano.2014.16

[b9] Tian J. *et al.* Topological insulator based spin valve devices: Evidence for spin polarized transport of spin-momentum-locked topological surface states. Solid State Commun. 191, 1 (2014).

[b10] Mellnik A. R. *et al.* Spin-transfer torque generated by a topological insulator. Nature 511, 449 (2014). 25056062 10.1038/nature13534

[b11] Fan Y. *et al.* Magnetization switching through giant spin–orbit torque in a magnetically doped topological insulator heterostructure. Nature Mater. 13, 699 (2014). 24776536 10.1038/nmat3973

[b12] Tang J. *et al.* Electrical Detection of Spin-Polarized Surface States Conduction in (Bi_0.53_Sb_0.47_)_2_Te_3_ Topological Insulator. Nano Lett. 14, 5423 (2014). 25158276 10.1021/nl5026198

[b13] Raghu S., Chung S. B., Qi X.-L. & Zhang S.-C. Collective Modes of a Helical Liquid. Phys. Rev. Lett. 104, 116401 (2010). 20366490 10.1103/PhysRevLett.104.116401

[b14] Di Pietro P. *et al.* Observation of Dirac plasmons in a topological insulator. Nature Nanotechnol. 8, 556 (2013). 23872838 10.1038/nnano.2013.134

[b15] Stauber T., Gómez-Santos G. & Brey L. Spin-charge separation of plasmonic excitations in thin topological insulators. Phys. Rev. B 88, 205427 (2013).

[b16] Crepaldi A. *et al.* Giant Ambipolar Rashba Effect in the Semiconductor BiTeI. Phys. Rev. Lett. 109, 096803 (2012). 23002871 10.1103/PhysRevLett.109.096803

[b17] Landolt G. *et al.* Disentanglement of Surface and Bulk Rashba Spin Splittings in Noncentrosymmetric BiTeI. Phys. Rev. Lett. 109, 116403 (2012). 23005655 10.1103/PhysRevLett.109.116403

[b18] Sakano M. *et al.* Strongly Spin-Orbit Coupled Two-Dimensional Electron Gas Emerging near the Surface of Polar Semiconductors. Phys. Rev. Lett. 110, 107204 (2013). 23521291 10.1103/PhysRevLett.110.107204

[b19] Eremeev S. V., Rusinov I. P., Nechaev A. I. & Chulkov E. V. Rashba split surface states in BiTeBr. New J. Phys. 15, 075015 (2013).

[b20] Landolt G. *et al.* Bulk and surface Rashba splitting in single termination BiTeCl. New J. Phys. 15, 085022 (2013).

[b21] Eremeev S. V., Nechaev I. A., Echenique P. M. & Chulkov E. V. Spin-helical Dirac states in graphene induced by polar-substrate surfaces with giant spin-orbit interaction: a new platform for spintronics. Sci. Rep. 4, 6900 (2014). 25365945 10.1038/srep06900 PMC4219157

[b22] Eremeev S. V. *et al.* Atom-specific spin mapping and buried topological states in a homologous series of topological insulators. Nature Commun. 3, 635 (2012). 22273673 10.1038/ncomms1638

[b23] Fu L. Hexagonal Warping Effects in the Surface States of Topological Insulator Bi_2_Te_3_. Phys. Rev. Lett. 103, 266801 (2009). 20366328 10.1103/PhysRevLett.103.266801

[b24] Bychkov Yu.A. & Rashba E. I. Properties of a 2D electron gas with lifted spectral degeneracy. JETP Lett. 39, 78 (1984).

[b25] Nechaev I. A., Echenique P. M. & Chulkov E. V. Inelastic decay rate of quasiparticles in a two-dimensional spin-orbit coupled electron system. Phys. Rev. B 81, 195112 (2010).

[b26] Qu D.-X., Hor Y. S., Xiong J., Cava R. J. & Ong N. P. Quantum Oscillations and Hall Anomaly of Surface States in the Topological Insulator Bi_2_Te_3_. Science 329, 821 (2010). 20671155 10.1126/science.1189792

[b27] Principi A., Carrega M., Asgari R., Pellegrini V. & Polini M. Plasmons and Coulomb drag in Dirac-Schrödinger hybrid electron systems. Phys. Rev. B 86, 085421 (2012).

[b28] Scharf B. & Matos-Abiague A. Coulomb drag between massless and massive fermions. Phys. Rev. B 86, 115425 (2012).

[b29] Saraga D. S. & Loss D. Fermi liquid parameters in two dimensions with spin-orbit interaction. Phys. Rev. B 72, 195319 (2005).

[b30] Das Sarma S., Hwang E. H. & Tse W.-K. Many-body interaction effects in doped and undoped graphene: Fermi liquid versus non-Fermi liquid. Phys. Rev. B 75, 121406(R) (2007).

