# Supplementary material for: New generation of two-dimensional spintronic systems realized by coupling of Rashba and Dirac fermions
Source: Sci Rep. 2015 Aug 4;5:12819. doi: 10.1038/srep12819 (PMC4523854; doi:10.1038/srep12819)
Supplement: Supplementary Information [file srep12819-s1.pdf]

Supplementary Information for
New generation of two-dimensional spintronic systems realized by coupling of Rashba and Dirac fermions

S. V. Eremeev, S. S. Tsirkin, I. A. Nechaev, P. M. Echenique, and E. V. Chulkov

(Dated: May 26, 2015)

S1. Details of DFT calculations

Electronic structure calculations were carried out within the density functional theory using the projector augmented-wave method[1] as implemented in the VASP code.[2,3] The exchange-correlation energy was treated using the generalized gradient approximation (GGA-PBE).[4] Relativistic effects, including spin-orbit interaction (SOI), were taken into account.

For bulk calculations, the lattice parameters of $PbSb_2Te_4$ were taken from Ref. 5. In this case, the rhombohedral unit cell is characterized by $a_{\rm rh} = 26.77$ a.u. and $\vartheta_{\rm rh} = 17.7105°$. Positions of the Pb, Te, and Sb atoms in the unit cell were obtained as a result of relaxation procedure optimizing the atomic positions at fixed unit-cell volume. This leads to the position of Pb at (0.000, 0.000, 0.000), Te^{I} at ($\pm\mu$, $\pm\mu$, $\pm\mu$) with $\mu = 0.13489$, Te^{II} at ($\pm\lambda$, $\pm\lambda$, $\pm\lambda$) with $\lambda = 0.28516$, and Sb at ($\pm\nu$, $\pm\nu$, $\pm\nu$) with $\nu = 0.42805$.

The $PbSb_2Te_4$ surface calculation was performed within slab model with using 42 atomic layer (six septuple layers) slab. A vacuum spacer of 20 Å was included to ensure a negligible interaction between neighboring slabs. The k-point mesh of $11 \times 11 \times 1$ was used for the Brillouin zone of the surface unit cell. The total-energy convergence was better than 1.0×10^{-6} eV. The same k-mesh and convergence criterion were used for freestanding BiTeI TL and BiTeI@$PbSb_2Te_4$ structures. The atomic positions at the BiTeI@$PbSb_2Te_4$ interfaces were obtained by structure optimization of the topmost layers with SOI included until forces affected them became less than 1.0×10^{-4} eV/Å. Since both materials, $PbSb_2Te_4$ and BiTeI, have layered structure being composed from septuple and triple layer blocks, respectively, separated by van der Waals spacings the total energy of the constructed BiTeI@$PbSb_2Te_4$ interfaces only slightly depends on the stacking order as well as on the BiTeI overlayer orientation. We have considered Te-Bi-I/Te-Sb-Te-Pb-Te-Sb-Te (TII) and I-Bi-Te/Te-Sb-Te-Pb-Te-Sb-Te (TTI) interfaces with A-C and A-A hexagonal stackings. The relaxed TII-AA and TTI-AA interfaces have almost the same total energy (the difference is of 0.5 meV). The energy gain for TII-AC with respect to TII-AA is small – it amounts of 15 meV only. In turn, the TTI-AC interface is just of 10 meV preferable with respect to TII-AC. In order to take into account the effect of dispersion interactions on interface relaxation, we use the van der Waals nonlocal correlation functional within the Grimme approach. The resulting relative energy differences differ from those obtained within GGA-PBE in few meV. Thus we conclude that all types of the considered interfaces can be realized in practice depending on the growth conditions. For TTI-AC and TII-AC cases the equilibrium interface distances are 2.81 and 2.86 Å, respectively, that is comparable with van der Waals Te-Te spacing in $PbSb_2Te_4$ (2.65 Å) and Te-I spacing in bulk BiTeI (2.97 Å). For TTI-AA and TII-AA interfaces the interlayer equilibrium distances are considerably larger: 3.78 and 3.84 Å, respectively, owing to that interfacial atoms of the trilayer are situated on-top of surface atoms of the substrate. At the same time, the Te-Te interatomic distances at AC and AA interfaces are almost the same: 3.79 and 3.78 Å, respectively. The Te-I interatomic distances at AC and AA interfaces also have similar values: 3.81 and 3.84 Å, respectively, and they are larger than Te-Te interatomic distances in TTI structures that correlate with longer Te-I in BiTeI than Te-Te in $PbSb_2Te_4$. The electron energy spectra of BiTeI@$PbSb_2Te_4$ interfaces calculated within the Grimme approach do not differ from those obtained within GGA-PBE.

In Figs. 1(a) and 1(b) the potential difference ΔV between electrostatic potentials of the relaxed heterostructure BiTeI@$PbSb_2Te_4$ and its spaced constituents (TI and TL) is shown for the A-C hexagonal stacking. As one can see, at the Te-Te interface the potential within the TL is shifted up by ~ 0.15 eV with respect to that in spaced TL, whereas the shift of the TL potential at the TII is negative and larger in absolute value (~ 0.20 eV) owing to stronger Te-I interaction. Such behavior of the potential should lead to a shift of BiTeI TL states up and down with respect to noninteracting case for TTI and TII, respectively.

S2. Model Hamiltonian description

The model Hamiltonian $\mathbf{H}$ written in the main text is diagonalized with the energies $E^{s\mu}_{\mathbf{k}} = (\widetilde{E}^{s}_{D\mathbf{k}} + \widetilde{E}^{s}_{R\mathbf{k}})/2 + \mu\sqrt{(\widetilde{E}^{s}_{D\mathbf{k}} - \widetilde{E}^{s}_{R\mathbf{k}})^2/4 + \Delta^2}$ and the wave functions $\Psi^{s\mu}_{\mathbf{k}} = \left(\psi^{D}_{s\mu\mathbf{k}}, \psi^{R}_{s\mu\mathbf{k}}\right)^T$, where two-component spinors have the form

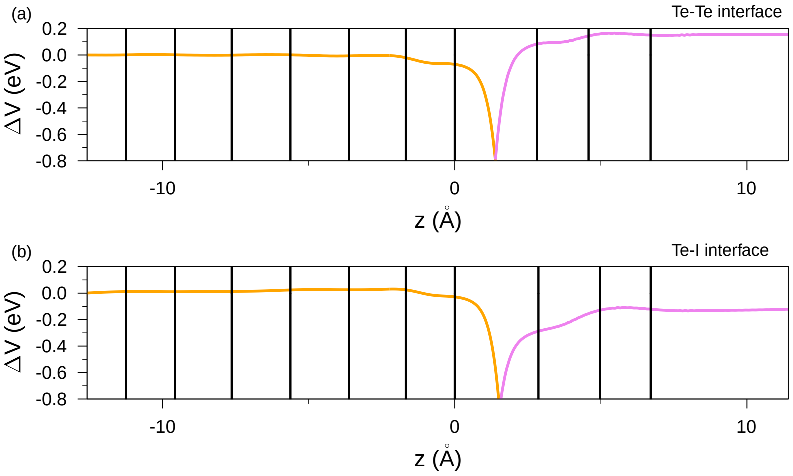

FIG. 1: Potential difference ΔV between total electrostatic potential of BiTeI@$PbSb_2Te_4$ heterostructure and potentials of $PbSb_2Te_4$ (orange line) and BiTeI (violet line) spaced slabs for (a) Te-Te and (b) Te-I interfaces with the A-C stacking; vertical lines mark positions of the atomic layers; layers at negative z are the TI substrate layers; layers at positive z are layers of BiTeI TL; $z = 0$ corresponds to the topmost layer of TI.

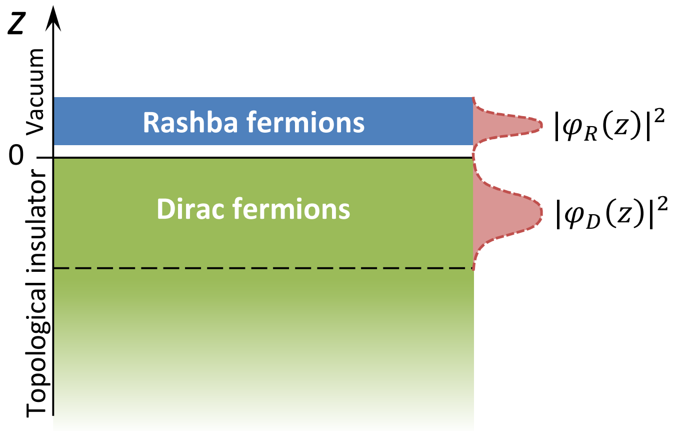

FIG. 2: Schematic representation of the geometry of the model Dirac-Rashba hybrid system.

of $\psi^i_{s\mu\mathbf{k}} = A^{s\mu}_{i\mathbf{k}}|s\rangle$. Here, i runs over the subsystems D and R, $s,\mu = \pm 1$, $\widetilde{E}^s_{i\mathbf{k}} = E^s_i + \frac{k^2}{2m^*_i} + s v_i k$, $v_R = \alpha_R$, $v_D = v_{D0}(1+\gamma k^2)$, $\sigma_z|s\rangle = s|s\rangle$. The normalization constants are: $A^{s\mu}_{R\mathbf{k}} = (\widetilde{E}^s_{D\mathbf{k}} - E^{s\mu}_{\mathbf{k}} - \Delta)/\beta^{s\mu}_{\mathbf{k}}$ and $A^{s\mu}_{D\mathbf{k}} = (\widetilde{E}^s_{R\mathbf{k}} - E^{s\mu}_{\mathbf{k}} - \Delta)/\beta^{s\mu}_{\mathbf{k}}$ with $\beta^{s\mu}_{\mathbf{k}} = \sqrt{(\widetilde{E}^s_{D\mathbf{k}} - E^{s\mu}_{\mathbf{k}} - \Delta)^2 + (\widetilde{E}^s_{R\mathbf{k}} - E^{s\mu}_{\mathbf{k}} - \Delta)^2}$. A desired form of the wavefunctions $\Psi^{s\mu}_{\mathbf{k}}$ in real space is $\Psi^{s\mu}_{\mathbf{k}}(\mathbf{r}_\parallel, z) = e^{i\mathbf{k}\mathbf{r}_\parallel}\sum_i \psi^i_{s\mu\mathbf{k}}\varphi_i(z)$, where $|\varphi_i(z)|^2$ characterizes the localization of the i-th system in the direction z perpendicular to the surface plane (see Fig. 2).

The best fitting for the TII-AC is reached, when we use the parameters $E_D = 0.13$, $E_R = 0.02$ eV $m_R = 0.3m_e$, $m_D = 0.5m_e$, $v_{D0} = 2$ eV Å, $\gamma = 150$ eV Å^3, $\alpha_R = 1.6$ eV Å, $\Delta = 0.05$ eV. The same parameters, but $E_R = 0.10$ eV, $E_D = 0.01$ eV, and $\Delta = 0.02$ eV are used to describe the TTI-AA.

With the model proposed, we can answer the question of what benefits (apart from the improvements mentioned in the main text) one has with depositing a TL of BiTeI on top of $PbSb_2Te_4$, and how the resulting Rashba-derived spin-helical 2D states differ from the Dirac-cone states in formation of dynamics of quasiparticles at the surface. We approximate the non-interacting response function of the Dirac-Rashba hybrid electron system under study by the form (for decoupled hybrid 2D systems see, e.g., Refs. 6–8)

$$\chi^0(\mathbf{q},\omega;z,z') = \sum_{i,j}|\varphi_i(z)|^2\chi^0_{ij}(\mathbf{q},\omega)|\varphi_j(z')|^2 \tag{1}$$

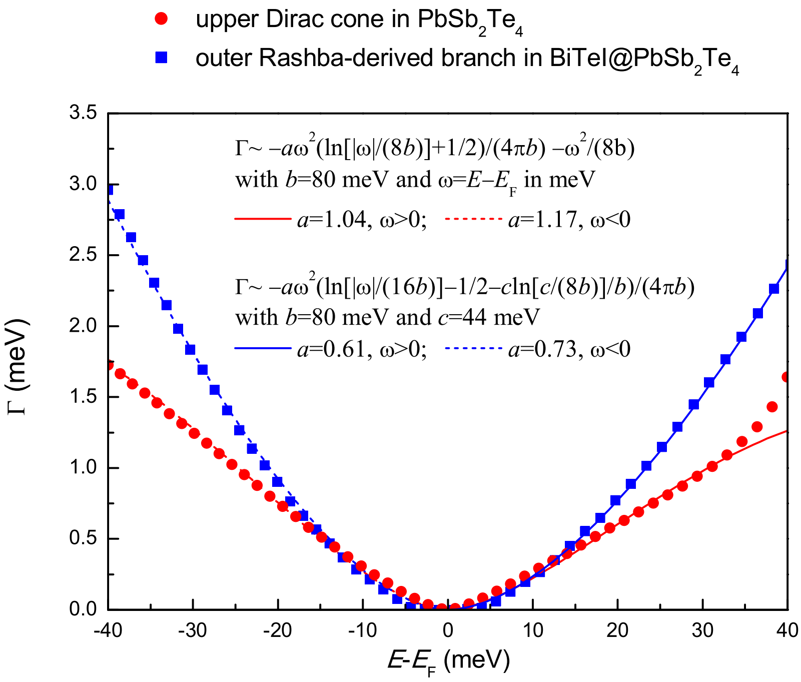

FIG. 3: Inelastic decay rate Γ of quasiparticles in the upper branch of the Dirac cone in the pristine $PbSb_2Te_4$ (red circles) and in the outer branch of the Rashba bands in BiTeI@$PbSb_2Te_4$ (blue squares) as a function of the excitation energy $\omega = E - E_F$ with the Fermi energy $E_F = 0.08$ eV (see Fig. 3 of the main text). Approximating curves are also shown (see the text).

with

$$\chi^0_{ij}(\mathbf{q},\omega) = \sum_{ss'\mu\mu'} \int \frac{d\mathbf{k}}{4\pi^2} \frac{\left(f^{\mathbf{k}}_{s'\mu'} - f^{\mathbf{k}+\mathbf{q}}_{s\mu}\right) F^{ss'}_{\mathbf{k},\mathbf{k}+\mathbf{q}} M^{ss'\mu\mu' ij}_{\mathbf{k},\mathbf{k}+\mathbf{q}}}{\omega + E^{s'\mu'}_{\mathbf{k}} - E^{s\mu}_{\mathbf{k}+\mathbf{q}} + i\eta},$$

where $F^{ss'}_{\mathbf{k},\mathbf{p}} = \frac{1}{2}\left[1 + ss'\cos(\phi_{\mathbf{p}} - \phi_{\mathbf{k}})\right]$, $f^{\mathbf{k}}_{s\mu}$ is the Fermi factor, and $M^{ss'\mu\mu' ij}_{\mathbf{k},\mathbf{p}} = A^{s\mu}_{i\mathbf{p}} A^{s'\mu'}_{i\mathbf{k}} A^{s'\mu'}_{j\mathbf{k}} A^{s\mu}_{j\mathbf{p}}$. In deriving Eq. (1), we neglected terms containing the products like $\varphi_D(z)\varphi^*_R(z)$ accounting for the overlap between the Dirac and Rashba subsystems along the z direction. Also, in calculations of χ^0_{ij} a finite value for the broadening parameter $\eta = 1$ meV was introduced. As a result, we arrive at the interacting response function within random phase approximation (RPA)

$$\chi(\mathbf{q},\omega; z, z') = \sum_{i,j} |\varphi_i(z)|^2 \chi_{ij}(\mathbf{q},\omega) |\varphi_j(z')|^2, \tag{2}$$

where $\chi_{ij}(\mathbf{q},\omega)$ in the contracted matrix form is given by $\boldsymbol{\chi}(\mathbf{q},\omega) = \left[\mathbf{I} - \boldsymbol{\chi}^0(\mathbf{q},\omega)\mathbf{V}(\mathbf{q})\right]^{-1} \boldsymbol{\chi}^0(\mathbf{q},\omega)$ with $\mathbf{I}$ being the identity matrix. The elements of the interaction $\mathbf{V}$ are defined as $V_{ij}(\mathbf{q}) = \int dz dz' |\varphi_i(z)|^2 v_c(\mathbf{q}; z, z') |\varphi_j(z')|^2$, where the function $v_c(\mathbf{q}; z, z')$ is the Coulomb interaction between a source charge at z' and a test charge at z as obtained from the Poisson equation with the z-dependent dielectric constant ε. The latter characterizes the medium, into which our 2D subsystems are submerged, and is thought to be 1 for $z > 0$ (in vacuum) and $\varepsilon_{\rm TI} = 53$ (see Sec. S3 below) for $z < 0$, where we place the semi-infinite topological insulator. In this case, we have (at any z')

$$\begin{aligned} v_c(\mathbf{q}; z > 0, z') &= \frac{2\pi}{q}\left(e^{-q|z-z'|} - \frac{\varepsilon_{\rm TI} - 1}{\varepsilon_{\rm TI} + 1} e^{-q(z+|z'|)}\right), \\ v_c(\mathbf{q}; z < 0, z') &= \frac{2\pi}{\varepsilon_{\rm TI} q}\left(e^{-q|z-z'|} + \frac{\varepsilon_{\rm TI} - 1}{\varepsilon_{\rm TI} + 1} e^{-q(|z|+|z'|)}\right). \end{aligned}$$

We consider the Rashba subsystem is located outside the TI in vacuum, while the Dirac electrons are hosted inside the TI (see Fig. 2). To gain insight into the properties of single-particle and collective excitations in the Dirac-Rashba hybrid system, we examine the electron energy loss function $g(\mathbf{q},\omega) = -\frac{2\pi}{q}{\rm Im}\left[\sum_{ij} \chi_{ij}(\mathbf{q},\omega)\right]$ and the real part (σ_1) of the optical conductivity $\sigma(\omega) = \lim_{q\to 0} \frac{i\omega}{q^2} \sum_{ij} \chi^0_{ij}(q,\omega)$.

For the quasiparticle decay rate $\Gamma_{s\mu}(\mathbf{k}) = 2|\mathrm{Im}\langle\Sigma_{s\mu}(\mathbf{k}, E^{s\mu}_{\mathbf{k}})\rangle|$ (inverse lifetime $\tau^{-1}_{s\mu}(\mathbf{k})$ caused by inelastic electron-electron scattering), we derived the on-shell matrix elements of the self-energy within the G_0W_0 approximation:

$$\mathrm{Im}\langle\Sigma_{s\mu}(\mathbf{k},\omega)\rangle = \mp \sum_{s'\mu' ij} \int \frac{d\mathbf{q}}{(2\pi)^2} F^{ss'}_{\mathbf{k},\mathbf{q}} f^{\mathbf{q}\pm}_{s'\mu'} \theta(\pm E^{s'\mu'}_{\mathbf{q}} \mp \omega) M^{s\mu s'\mu' ij}_{\mathbf{q},\mathbf{k}} \mathrm{Im} W^0_{ij}(\mathbf{k}-\mathbf{q}, \omega - E^{s'\mu'}_{\mathbf{q}}), \tag{3}$$

where the upper sign corresponds to $\omega < E_F$ and the lower sign to $\omega > E_F$, $f^{\mathbf{q}+}_{s'\mu'} = f^{\mathbf{q}}_{s'\mu'}$, $f^{\mathbf{q}-}_{s'\mu'} = 1 - f^{\mathbf{q}}_{s'\mu'}$, $\theta(x)$ is the step function, and the screened interaction $W^0_{ij}(\mathbf{q},\omega) = V_{ij}(\mathbf{q}) + [\mathbf{V}(\mathbf{q})\boldsymbol{\chi}(\mathbf{q},\omega)\mathbf{V}(\mathbf{q})]_{ij}$.

Both for the pure Dirac system (the pristine $PbSb_2Te_4$) and for the Dirac-Rashba hybrid one ($BiTeI@PbSb_2Te_4$), we analyzed the inelastic decay rate of quasiparticles in the 2D states that reside within the energy interval corresponding to the gap highlighted by the yellow stripe in Fig. 3 of the main text. We put the Fermi level at the center of this interval ($E_F = 0.08$ eV in the mentioned main-text figure). The obtained results are shown in Fig. 3. First of all, we note that as compared with the pure Dirac fermions in the hybrid system the inelastic decay rate is slightly smaller for excitation energies $|E - E_F| \lesssim 15$ meV and tends to be somewhat bigger beyond these confines. Second, within the energy interval we are interested in, the results on $\Gamma_{s\mu}$ for the hybrid system can be well approximated by the functional dependence of the inelastic decay rate on the excitation energy as derived for Rashba fermions in Ref. 9. Actually, as seen in Fig. 3, the decay rate of quasiparticles in the outer Rashba-derived branch is nicely reproduced by $\propto \frac{\omega^2}{4\pi b}\left[\ln\left(\frac{\omega}{16b}\right) - \frac{1}{2} - \frac{c}{b}\ln\left(\frac{c}{8b}\right)\right]$, where b and c, in fact, stand for the Fermi and Rashba energy, respectively, as measured from the degeneracy point. For the pure Dirac system, the dependence[10] $\propto \frac{\omega^2}{4\pi b}\left[\ln\left(\frac{\omega}{8b}\right) + \frac{1}{2}\right]$ can be used. However, in this case, as is shown in the figure, an additional term $-\frac{\omega^2}{8b}$ should be taken into account to cover properly the whole energy interval. A notable deviation of the calculated inelastic rate of electrons from the approximating curve at $E - E_F \gtrsim 30$ meV is a plasmon-related effect possible due to the non-linear dispersion $\widetilde{E}^s_{D\mathbf{k}}$.

S3. Dielectric constant

Similar to Ref. 11, in our *ab initio* calculations of the dielectric tensor and underlying DFT band structure of the bulk $PbSb_2Te_4$, we employ the full-potential linearized augmented plane-wave (FLAPW) method as implemented in the FLEUR code[12] within the GGA of Ref. 4 for the exchange-correlation functional. The ground-state calculations were performed with the use of a plane-wave cutoff of $k_{max} = 4.0$ bohr^{-1}, an angular momentum cutoff of $l_{max} = 12$ for Pb and $l_{max} = 10$ for Sb and Te, and a $7 \times 7 \times 7$ Γ-centered **k**-point sampling of the BZ. The FLAPW basis has been extended by conventional local orbitals[13,14] to treat semi-core d-states (5d for Pb and 4d for Sb and Te). The energy cutoff between core and valence states was put at -1.8 Ha, what corresponds to 108 valence electrons in the considered energy window in a rhombohedral $PbSb_2Te_4$. In order to more accurately describe high-lying unoccupied states,[15] one local orbital per angular momentum up to $l = 3$ was included for each atom. The Fermi level was placed in the middle of the band gap.

The dielectric tensor is found by using the SPEX code with inclusion of spin-orbit interaction.[16,17] The dielectric matrix was evaluated within the random-phase approximation and represented with the use of the mixed product basis,[16,18] where we chose an angular momentum cutoff in the muffin-tin spheres of 4 and a linear momentum cutoff of 3.5 bohr^{-1}. For the bulk $PbSb_2Te_4$, we have found that the dielectric constant $\varepsilon_{\mathrm{TI}} = 53$ being an average over the components $\varepsilon_{\perp c} = 58$ and $\varepsilon_{\parallel c} = 41$ of the dielectric tensor. We used this value of $\varepsilon_{\mathrm{TI}}$ in our calculations of the response function described in Sec. S2.

1 P.E. Blöchl, Phys. Rev. B **50**, 17953 (1994).
2 G. Kresse and J. Furthmüller, Phys. Rev. B **54**, 11169 (1996).
3 G. Kresse and D. Joubert, Phys. Rev. B **59**, 1758 (1999).
4 J.P. Perdew, K. Burke, and M. Ernzerhof, Phys. Rev. Lett. **77**, 3865 (1996); J.P. Perdew, K. Burke, and M. Ernzerhof, Phys. Rev. Lett. **78**, 1396(E) (1997).
5 L.E. Shelimova, O.G. Karpinskii, T.E. Svechnikova, I.Y. Nikhezina, E.S. Avilov, M.A. Kretova, V.S. Zemskov, Inorg. Mater. **44**, 371 (2008).
6 A. Principi, M. Carrega, R. Asgari, V. Pellegrini, and M. Polini, Phys. Rev. B **86**, 085421 (2012).
7 B. Scharf and A. Matos-Abiague, Phys. Rev. B **86**, 115425 (2012).
8 A. C. Balram, J. A. Hutasoit, and J. K. Jain, Phys. Rev. B **90**, 045103 (2014).
9 S. Das Sarma, E. H. Hwang, and W.-K. Tse, Phys. Rev. B **75**, 121406(R) (2007).
10 D.S. Saraga and D. Loss, Phys. Rev. B **72**, 195319 (2005).

[11] I.A. Nechaev, R.C. Hatch, M. Bianchi, D. Guan, C. Friedrich, I. Aguilera, J.L. Mi, B.B. Iversen, S. Blügel, Ph. Hofmann, and E.V. Chulkov, Phys. Rev. B **87**, 121111(R) (2013).
[12] [http://www.flapw.de].
[13] D. Singh, Phys. Rev. B **43**, 6388 (1991).
[14] E. Sjöstedt, L. Nordström, and D. J. Singh, Solid State Commun. **114**, 15 (2000).
[15] E. E. Krasovskii, Phys. Rev. B **56**, 12866 (1997).
[16] C. Friedrich, S. Blügel, and A. Schindlmayr, Phys. Rev. B **81**, 125102 (2010).
[17] R. Sakuma, C. Friedrich, T. Miyake, S. Blügel, and F. Aryasetiawan, Phys. Rev. B **84**, 085144 (2011).
[18] T. Kotani and M. van Schilfgaarde, Solid State Commun. **121**, 461 (2002).
